# UV-Assisted Charge Neutralization for Reliable XPS Measurements on Insulating Materials

**DOI:** 10.3390/ma18133051

**Published:** 2025-06-27

**Authors:** Lei Zhu, Xuefeng Xu

**Affiliations:** School of Technology, Beijing Forestry University, Beijing 100083, China; zhulei2018@bjfu.edu.cn

**Keywords:** XPS, surface charging, charge neutralization, photoelectron, UV

## Abstract

When analyzing insulator surfaces using X-ray photoelectron spectroscopy (XPS), spectral shifts and deformations often arise due to surface charging. Although neutralization techniques have been widely adopted to achieve reliable XPS measurements, their effectiveness remains limited, highlighting the need for innovative neutralization strategies. Here, ultraviolet (UV) light irradiation was introduced into XPS measurements. Although it was still impossible to perfectly eliminate the charging effect, stable XPS spectra with reduced and consistent spectral shifts, as well as minimal deformation and broadening, were successfully obtained. Our findings demonstrate that UV light irradiation not only significantly mitigates the intensity of surface charging but also markedly enhances both its temporal stability and spatial uniformity during XPS measurements. Further investigation reveals that the suppression of charging is primarily attributed to the adsorption of UV-excited photoelectrons onto the X-ray-irradiated region. This innovative neutralization method, termed UV-assisted neutralization in this article, was found to be at least as effective as and even superior in maintaining sample integrity to the most commonly used dual-beam charge neutralization, and therefore is expected to become a promising alternative for addressing the charging issues in XPS measurements.

## 1. Introduction

X-ray photoelectron spectroscopy (XPS) is currently one of the most widely used surface analytical techniques, providing valuable information about the chemical states, bonding structure, and elemental composition of the sample surface [1,2,3,4,5,6]. During XPS measurements, photoelectrons are continuously excited from the surface region irradiated by the X-ray beam. For poorly conductive samples, the loss of electrons cannot be replenished in a timely manner, leading to the accumulation of positive charges on the surface. This phenomenon, known as surface charging, disrupts charge neutrality and can cause photoelectron spectra to shift toward higher binding energy (BE) or even distort their shapes, thereby rendering the spectral data misleading [7,8,9,10]. Consequently, effective neutralization of surface charges is essential for obtaining accurate and reliable XPS spectra.

At present, the most widely used method for charge neutralization in XPS involves the use of an electron flood gun or a combination of electron and ion beams [11,12,13,14,15]. While this approach can stabilize the charging state on the sample surface, it often results in under- or over-compensation, leading to a nonzero electric potential on the sample surface and a consequent shift in the entire XPS spectrum along the binding energy axis [16,17]. To correct for this shift, characteristic peaks with known binding energies (BEs) of intrinsic elements of the material itself [18], the ubiquitous adventitious carbon layer (AdC) [19,20], or externally added elements such as implanted argon [21] or sputter-deposited gold [22,23,24] have been employed as binding energy references. Among these, referencing the BE scale to the C 1s spectra of AdC has been incorporated into both ASTM and ISO standards [25,26].

In addition to the electron flood gun, other neutralization techniques have also been employed to address charging issues in XPS measurements. These include allowing charges on the irradiated surface to find a pathway to the ground through heating [15,27,28] or illumination [28,29,30,31], as well as neutralizing positive charges on the sample surface using electrons produced during the photoionization of ambient gas in the chamber via ultraviolet (UV) [32] or X-ray irradiation [33]. While these methods offer feasible solutions for charge minimization, the ISO standard acknowledges that there is currently no universally applicable method for charge neutralization in XPS measurements [26]. Existing neutralization techniques cannot completely eliminate charging issues, for instance, non-uniform charging remains unresolved in certain cases [7,24,34,35,36]. Moreover, prolonged exposure to dual-beam flood sources may induce a reduction in metal ions and a loss of carbon atoms on the sample surfaces [13,37]. Heating or illumination methods are effective only for a limited subset of insulators exhibiting appreciable pyroconductivity or photoconductivity. Therefore, the development of new neutralization technologies remains essential for obtaining accurate XPS spectra.

This study shows that UV light irradiation significantly mitigates surface charging on bulk insulators exposed to X-rays under ultra-high vacuum (UHV) conditions. UV light not only markedly reduces the magnitude of surface charging but also enhances both the temporal stability and spatial uniformity of the charging during XPS measurements. The effectiveness of UV light as a charge neutralization method was systematically investigated and compared with the conventional dual-beam charge neutralization technique. Furthermore, the underlying mechanisms of UV light neutralization were explored. The results indicate that the neutralization effect is primarily attributed to the adsorption of UV-excited photoelectrons onto the X-ray-irradiated region.

## 2. Materials and Methods

The photoelectron spectra were obtained using photoelectron spectroscopy (PES, PHI-5000 VersaProbe III, JAPAN). A monochromatic Al Kα X-ray line (hν = 1486.6 eV) with a line width of 0.85 eV was employed as the X-ray source, while the He I line (hν = 21.2 eV) and He II line (hν = 40.8 eV) served as UV sources. These three light sources are also typically used for XPS and UPS, respectively. All experiments were conducted in an UHV chamber with a base pressure of approximately 10^−8^ mbar. A Spherical Capacitor Analyzer was utilized to measure the energy of the photoelectrons. The energy scale and its linearity were automatically calibrated by referencing the peak positions of the Ag 3d_5/2_, Au 4f_7/2_, and Cu 2p_3/2_ lines. The UPS spectra were acquired with a pass energy of 2.6 eV and a step size of 0.02 eV.

The samples employed in this study consist of three types of bulk insulators: round disks of α-Al_2_O_3_ crystal (0001) and SiO_2_ glass, each with a diameter of 30 mm and a thickness of 2 mm, as well as square polyethylene terephthalate (PET) slices with a side length of 25 mm and a thickness of 0.5 mm. Fresh PET slices were used to minimize the potential for aging or contamination. Bulk Al_2_O_3_ and SiO_2_ are frequently employed as typical highly insulating materials to demonstrate charge-induced distortions in XPS spectra [7], while PET is widely adopted as a test sample in XPS measurements [15]. All samples were mounted on the sample holder using double-sided carbon tape. To evaluate the effectiveness of UV irradiation as a charge neutralization method, its performance was compared with the standard dual-beam charge neutralization technique. The dual-beam method employs a cold cathode electron flood source with an emission current of 20 μA and a low-energy ion source simultaneously. To optimize the signal-to-noise ratio in XPS measurements, the take-off angle (TOA), defined as the angle between the centerline of the analyzer detector and the plane of the sample, was set at 90° for measurements with UV irradiation and 45° for those with dual-beam charge neutralization.

## 3. Results and Discussion

### 3.1. UV-Assisted Charge Neutralization

**Figure 1:**
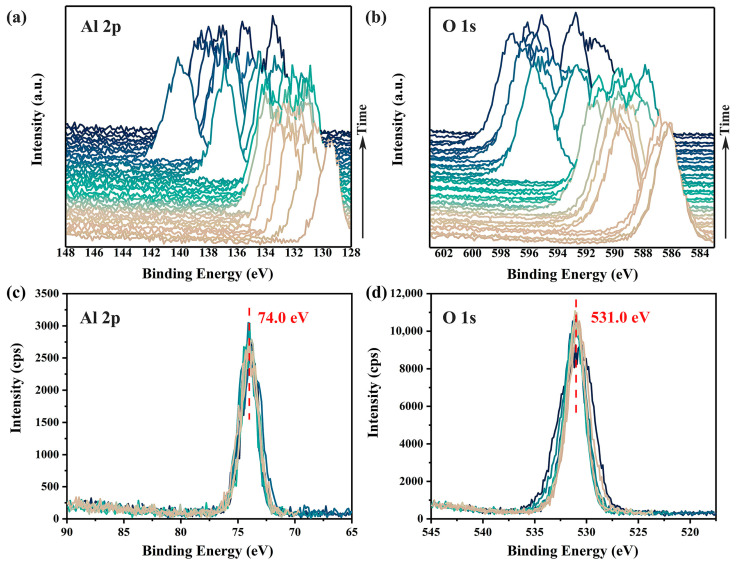
Time-dependent charging effects on the Al 2p (**a**) and O 1s (**b**) XPS spectra of α-Al_2_O_3_ surface without any charge compensation. Corrected spectra are shown in (**c**,**d**). Variations in spectra shapes of Al 2p (**c**) and O 1s (**d**) highlight differential charging across the irradiated surface. XPS spectra were acquired using a pass energy of 69 eV and a scanning step of 0.125 eV, with a total time spent of about 45 min.

To demonstrate the charging effects induced by a focused monochromatic X-ray, a 100 μm X-ray beam with a beam power of 25 W was used to continuously irradiate the surface of an α-Al_2_O_3_ crystal (0001). Two XPS spectra, covering the binding energy ranges of 128–148 eV (Al 2p spectra) and 583–603 eV (O 1s spectra), were measured sequentially and repeatedly without any charge neutralization. As shown in Figure 1a,b, the accumulation of positive charges on the sample surface due to irradiation led to spectral shifts of approximately 60 to 70 eV towards higher binding energy. Even after 40 rounds of measurements, both the positions of the peaks and the distance between them continued to exhibit significant and irregular fluctuations, indicating that the surface charging had not yet stabilized. Additionally, the pronounced differences in spectra shapes across different measurements, as shown in Figure 1c,d, reveal substantial non-uniform surface charging (i.e., differential charging [7]) within the irradiated surface region.

**Figure 2:**
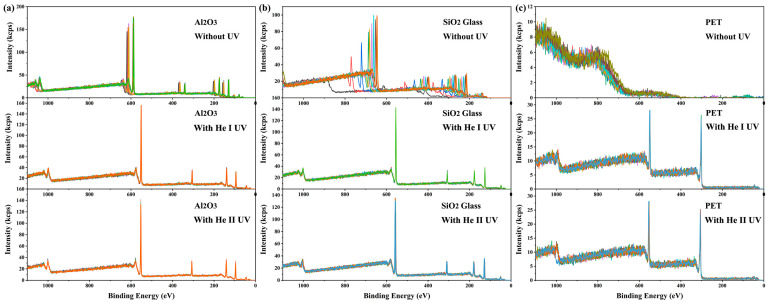
Ten consecutive measurements of full XPS spectra on (**a**) α-Al_2_O_3_ (0001) surface, (**b**) SiO_2_ glass surface, and (**c**) PET surface. The results of each measurement are shown in spectra of different colors. All measurements were carried out in the absence of dual-beam charge neutralization. Spectra were acquired with a pass energy of 280 eV and a scanning step of 1 eV.

Similar to X-ray irradiation, UV light irradiation can also cause the accumulation of positive charges on the irradiated surface and lead to a binding energy shift [38,39,40]. Intuitively, one might expect that the simultaneous irradiation of X-rays and UV light on the same surface region would exacerbate the charging effect. However, contrary to this expectation, it was found that the addition of UV light irradiation dramatically mitigated the charging on the X-ray-irradiated surface (see Figure 2). Without UV irradiation or dual-beam charge neutralization, the charging-induced binding energy shifts in XPS spectra from 10 consecutive measurements on α-Al_2_O_3_ crystal and SiO_2_ glass were time-dependent, ranging between 55 and 80 eV and between 110 and 330 eV, respectively (see Figure 2a,b). The charging effect on PET surfaces was even more severe, rendering it impossible to obtain meaningful XPS spectra (see Figure 2c). Surprisingly, under He I irradiation (lamp power 65 W), the spectral shifts and their fluctuations throughout 10 consecutive measurements were reduced to 20.9 eV and 0.09 eV for α-Al_2_O_3_, 22.6 eV and 0.12 eV for SiO_2_ glass, and 17.5 eV and 0.12 eV for PET, respectively. Under He II irradiation (lamp power 65 W), the corresponding values were 22.2 eV and 0.16 eV for α-Al_2_O_3_, 24.9 eV and 0.41 eV for SiO_2_ glass, and 21.2 eV and 0.33 eV for PET (see Figure 2). These results demonstrate that UV irradiation significantly reduces the magnitude and enhances the stability of surface charging induced by X-ray irradiation. This phenomenon is termed here as UV-assisted neutralization. The results show that the He I source provided more effective neutralization than the He II source. Additionally, since the discharge pressure required for He II UV excitation is much lower than that for He I UV excitation (approximately 5 × 10^−3^ mbar and 3 × 10^−2^ mbar, respectively), the stability of the light source is lower during long-term measurements, and light quenching may even occur. Such instability may lead to inconsistent neutralization performance (blue curve, bottom of Figure 2c). Therefore, He I UV light was selected for subsequent studies.

### 3.2. Effectiveness of UV-Assisted Neutralization

**Figure 3:**
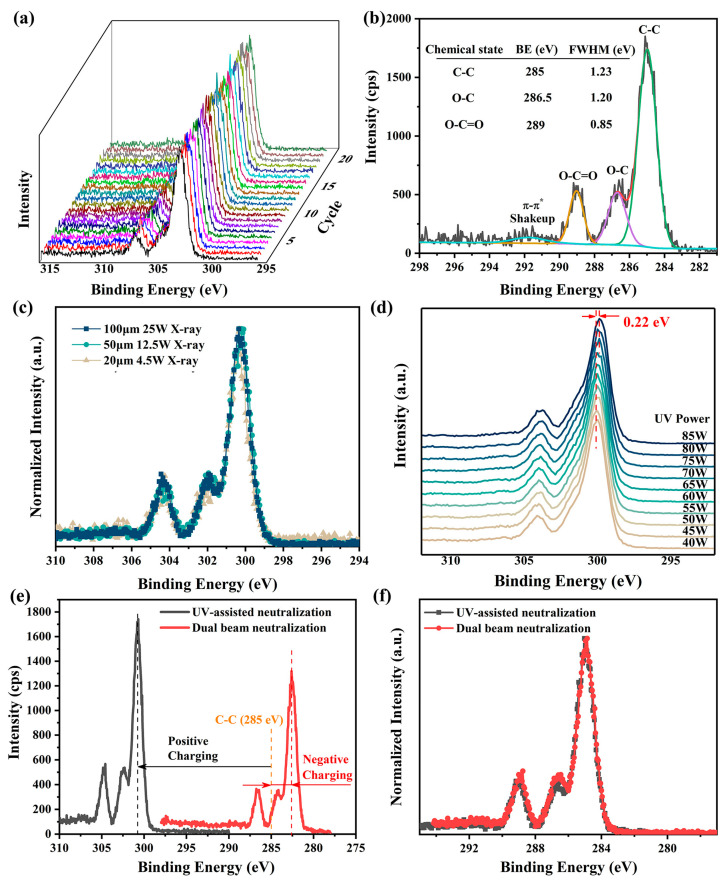
Neutralization effectiveness of He I irradiation (lamp power 65 W, except for experiments in (**d**)) in XPS measurements on PET surfaces. (**a**) Twenty consecutive measurements of narrow XPS spectra of C 1s spectra with UV-assisted neutralization. (**b**) Charge-corrected C 1s XPS spectra measured with UV-assisted neutralization, along with peak-fitted results. (**c**) Comparison of normalized, non-charge-corrected C 1s XPS spectra obtained under different X-ray settings in the presence of UV-assisted neutralization. (**d**) Peak positions of C 1s XPS spectra under He I UV irradiation with different powers, measured with a pass energy of 69 eV and a step size of 0.125 eV. (**e**) Uncorrected C 1s XPS spectra measured with UV-assisted neutralization and with dual-beam charge neutralization. (**f**) Comparison of normalized, charge-corrected C 1s XPS spectra obtained using the two neutralization methods. A 100 μm X-ray with a beam power of 25 W was used for all measurements, except those in (**c**). A pass energy of 26 eV and a step size of 0.05 eV were used for all XPS measurements, except those in (**d**).

The performance of UV-assisted neutralization was evaluated using narrow C 1s XPS spectra of PET, an insulating polymer with well-established XPS spectra [15,17,41]. The spectra were continuously measured 20 times with a pass energy of 26 eV and a step size of 0.05 eV under He I UV light irradiation (see Figure 3a). The standard deviation of the charging-induced surface potential, calculated based on the positions of the C-C XPS spectra, was found to be no more than 0.03 V, indicating highly stable surface charging. Additionally, the Full Width at Half Maximum (FWHM) of the O-C=O XPS spectrum, which is not easily affected by structural variations in PET [41], was measured to be approximately 0.85 eV (see Figure 3b). This value matches the line width of the X-ray source, suggesting nearly homogeneous charging across the measured region. These findings demonstrate that UV light irradiation significantly enhances both the temporal stability and spatial uniformity of surface charging in XPS.

In the presence of UV-assisted neutralization, the uncorrected XPS spectra obtained on the PET surface under various X-ray settings, including different beam sizes and powers, are nearly identical (see Figure 3c). This consistency underscores the suitability of UV-assisted neutralization for a wide range of X-ray configurations in XPS measurements. Further investigations reveal that significant changes in UV lamp power (from 40 W to 85 W) induce only a minor shift (0.22 eV in total) in the XPS spectra with no alteration in spectra shape (see Figure 3d). Given that fluctuations in UV lamp power during measurements, typically caused by variations in He gas pressure, are usually within 1–2 W in our experiments, the resulting shifts in XPS spectra are often less than 0.01 eV and can thus be neglected.

Although UV-assisted neutralization often results in under-neutralization, leading to a higher charging-induced surface potential compared to dual-beam charge neutralization (see Figure 3e), the corrected and normalized XPS spectra obtained using both methods are virtually indistinguishable (see Figure 3f). This means that the UV-assisted neutralization is at least as effective as the dual-beam charge neutralization. Moreover, during continuous measurements over 25 min, the shape and intensity of the C 1s spectra remained virtually unchanged (see Figure 3a), indicating that UV-assisted neutralization causes minimal damage to sample surfaces and is to some extent superior to dual-beam neutralization in preserving sample integrity [13,37]. Collectively, these results suggest that UV-assisted neutralization holds significant potential as a viable alternative for addressing charging issues in XPS measurements.

### 3.3. Neutralization Mechanisms of UV Light Irradiation

**Figure 4:**
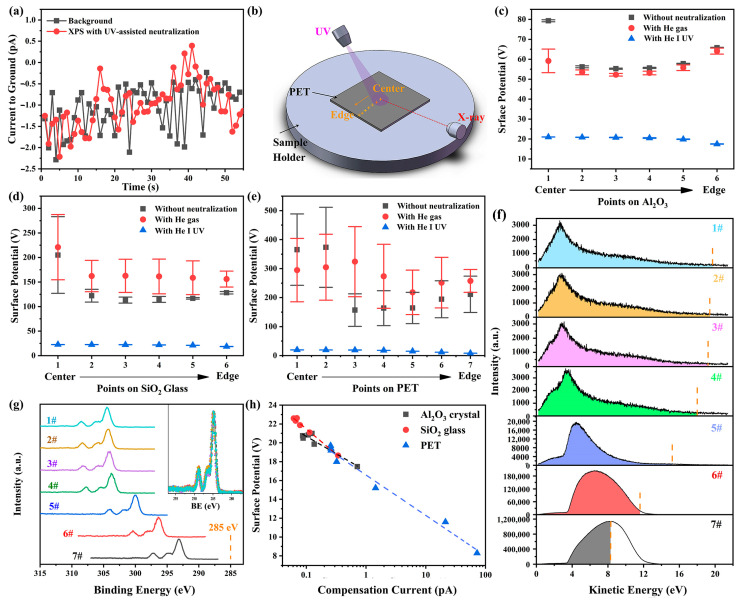
(**a**) Current measured from the sample holder to the ground during XPS measurements with UV-assisted neutralization and its comparison with the background level. (**b**) Schematic illustration of XPS measurements under three conditions: without any neutralization, with helium gas present but the UV lamp off, and with UV irradiation. Measurements were performed at different points arranged sequentially from the center to the edge of sample surfaces. Charging-induced surface potential and its standard deviation at different measurement points on (**c**) α-Al_2_O_3_ crystal, (**d**) SiO_2_ glass, and (**e**) PET, obtained from five sets of XPS measurements. (**f**) UPS spectra obtained at measurement points on PET surface in the absence of X-ray irradiation. The vertical dashed lines indicate the surface potential of the X-ray-irradiated region in the corresponding XPS measurements, while the shaded regions represent the compensating electrons. (**g**) Narrow C 1s spectra obtained at measurement points on PET surface under UV irradiation. The inset shows these spectra after charge correction and intensity normalization. (**h**) The relationship between the surface potentials on the X-ray-irradiated region and the compensation currents on surfaces of α-Al_2_O_3_ crystal (0001), SiO_2_ glass and PET.

Our findings imply that the electron loss on the sample surface caused by X-ray irradiation can be partially compensated by the introduction of UV light irradiation. The potential increase in surface conductivity induced by UV irradiation [29] is unlikely to explain the observed charge neutralization because the size and thickness of the samples used here far exceed the spot size and penetration depth of the lights in PES, preventing accumulated charges on the irradiated surface from finding a pathway to the ground. To further substantiate this point, the current from the sample holder to the ground was measured using a Keithley 6517B electrometer during the XPS measurements with UV-assisted neutralization. The measured current was nearly identical to the background level (see Figure 4a), indicating that the compensating electrons do not originate from the ground.

Another possible mechanism involves the increase in gas molecules in the chamber due to the helium UV source. Research in near-ambient pressure XPS (NAP-XPS) has demonstrated that UV irradiation can photoionize the ambient gas, thereby generating additional ambient electrons to neutralize positive charges on sample surfaces [32]. Consequently, the neutralization efficacy depends on gas species and pressure. To evaluate whether this mechanism dominates in UV-assisted neutralization applied to high-vacuum XPS measurements, experiments were conducted under three conditions: without any neutralization, with helium gas present but the UV lamp off, and with UV irradiation. Measurements were taken at different points arranged sequentially from the center to the edge of Al_2_O_3_, SiO_2_, and PET surfaces (see Figure 4b). When helium gas was introduced, the chamber pressure rose from 3.4 × 10^−8^ mbar to 4.6 × 10^−8^ mbar. The calculated charging-induced surface potentials (see Figure 4c–e) demonstrate that UV irradiation effectively reduces surface charging on all the surfaces, whereas merely increasing the gas molecules in the chamber worsens the charging condition rather than alleviating it. Therefore, the UV-induced increase in chamber pressure and the potential gas ionization are not the primary mechanisms for UV-assisted neutralization.

The XPS spectra under UV irradiation exhibit nearly identical shapes across all measurement points (see inset in Figure 4g). However, the surface potential on the X-ray-irradiated areas, indicated by energy shifts in the XPS spectra, exhibits a pronounced position dependence: it remains consistent in the central region (points 1–3 in Figure 4g) but gradually decrease as the measurement point approaches the sample edge (from point 4 to point 7 in Figure 4g). Exploring the underlying reasons for this spatial variation in surface potential could help elucidate the mechanisms of neutralization. The consistent shape of the XPS spectra across different points suggests that the position dependence of the surface potential is unlikely due to surface defect distribution or sample inhomogeneity. This conclusion is further supported by the inconsistent spatial trends in surface potential between the no neutralization and UV irradiation cases (Figure 4c–e). Additionally, enhanced conductivity of sample edges due to UV irradiation is also ruled out. This is evidenced by two factors: (1) PET slices were placed to prevent UV light from reaching the sample edges, and (2) if such enhanced conductivity formed a conductive path to ground, the energy shift differences in XPS spectra at positions 4–7 would not be significant.

To investigate the position dependence of surface potentials, UPS spectra were also measured at corresponding points in the absence of X-ray. The UPS spectra exhibit a similar position dependence to the XPS spectra. This discrepancy arises from variations in the actual UV-irradiated surface region at different positions. In the central region (points 1–3), where the UV beam irradiates entirely within the sample surface, the UPS spectra are nearly identical. However, as the measurement points approach the edge (points 4–7), an increasing portion of the UV beam irradiates the metal stage instead of the sample due to the larger UV beam diameter (~3mm) compared to the X-ray beam (~100 μm), leading to a significant increase in photoelectron yield and noticeable changes in the energy distribution of photoelectrons (see Figure 4f).

Our previous works have demonstrated that the charging on insulator surfaces irradiated by UV light alone can reach a steady state extremely rapidly (within 1 s). Both the magnitude and fluctuation of the charging-induced surface potential are minimal, approximately 2 V and within 0.02 V, respectively [38]. The rapid establishment of this satisfactory balance between electron emission and compensation may be attributed to the significantly larger UV spot size compared to the X-ray spot, enabling the UV-irradiated area to replenish electrons more efficiently from the surrounding environment.

Since the surface potential of the UV-irradiated region is considerably lower than that of the X-ray-irradiated surface, these UV photoelectrons, which have kinetic energies lower than the surface potential of the X-ray-irradiated region (referred to as the compensating electrons), will be attracted to the X-ray-irradiated surface by the electric force after they are emitted from the UV-irradiated region, thereby replenishing the electrons lost at the X-ray-irradiated surface. It is evident that as measurement positions approach the sample edge, progressively more of the UV beam irradiates the metal sample holder rather than the sample surface, leading to a significant increase in photoelectron yield (see Figure 4f), and a higher UV photoelectron yield results in a greater compensation current (as determined by integrating the compensating electrons in the UPS spectrum), which in turn enhances the neutralization effect and reduces the surface potential on the X-ray-irradiated area (see Figure 4h). The consistent and strong correlation between surface potential and compensation current observed across all materials studied here strongly suggests that the adsorption of UV photoelectrons onto the X-ray-irradiated region is the predominant mechanism underlying the observed UV-assisted neutralization.

## 4. Conclusions

Here, we present a UV-assisted charge neutralization technique for XPS analysis of insulating surfaces under UHV conditions. This study demonstrates that ultraviolet (UV) light irradiation can effectively mitigate charging issues in the X-ray-irradiated region, significantly reducing the charging-induced surface potential while improving both the temporal stability and spatial uniformity of surface charging. The UV-assisted neutralization is shown to be compatible with a wide range of X-ray settings in XPS measurements, with negligible spectral shifts caused by fluctuations in UV lamp power during measurements.

Furthermore, the underlying mechanism of this neutralization has been systematically investigated. It has been confirmed that neither the increase in surface conductivity nor the rise in chamber pressure or the potential gas ionization due to UV light introduction is the primary cause of the observed neutralization. Instead, the strong correlation between the surface potential of the X-ray-irradiated region and the yield of UV-excited photoelectrons suggests that the adsorption of UV-excited photoelectrons from the UV-irradiated surface onto the X-ray-irradiated region is likely the dominant mechanism of the neutralization. During XPS measurements, UV irradiation functions as an “electron pump”, drawing electrons from the UV-irradiated surface and delivering them to the X-ray-irradiated region.

While further investigation is warranted to determine the practical efficacy of UV-assisted neutralization on complex surfaces, such as heterogeneous or composite materials, and potential UV-induced surface chemical alterations represent a limitation, the experimental measurements on three representative materials (Al_2_O_3_ crystal, SiO_2_ glass, and PET) in this article demonstrate that this approach effectively mitigates charging effects across most insulating surfaces. It provides a highly stable and uniform charging surface. Its performance is at least comparable to that of dual-beam charge neutralization. Additionally, this technique causes minimal damage to sample surfaces, making it superior to dual-beam neutralization in preserving sample integrity. These suggest that the UV-assisted neutralization technique presented here could serve as a viable alternative for addressing charging issues in XPS measurements.

## Data Availability

The original contributions presented in the study are included in the article. Further inquiries may be directed to the corresponding author.

## Abbreviations

The following abbreviations are used in this manuscript:

XPS	X-ray photoelectron spectroscopy
UV	ultraviolet
UPS	ultraviolet photoelectron spectroscopy
BE	binding energy
AdC	adventitious carbon
UHV	ultra-high vacuum
TOA	take-off angle
PET	polyethylene terephthalate
FWHM	full width at half maximum
